# Flagellin Encoded in Gene-Based Vector Vaccines Is a Route-Dependent Immune Adjuvant

**DOI:** 10.1371/journal.pone.0148701

**Published:** 2016-02-04

**Authors:** Hamada F. Rady, Guixiang Dai, Weitao Huang, Judd E. Shellito, Alistair J. Ramsay

**Affiliations:** 1 Department of Microbiology, Immunology and Parasitology, Louisiana State University Health Sciences Center, New Orleans, Louisiana, United States of America; 2 Department of Medicine, Louisiana State University Health Sciences Center, New Orleans, Louisiana, United States of America; 3 Louisiana Vaccine Center, Louisiana State University Health Sciences Center, New Orleans, Louisiana, United States of America; University of Massachusetts Medical School, UNITED STATES

## Abstract

Flagellin has been tested as a protein-based vaccine adjuvant, with the majority of studies focused on antibody responses. Here, we evaluated the adjuvant activity of flagellin for both cellular and humoral immune responses in BALB/c mice in the setting of gene-based immunization, and have made several novel observations. DNA vaccines and adenovirus (Ad) vectors were engineered to encode mycobacterial protein Ag85B, with or without flagellin of *Salmonella typhimurium* (FliC). DNA-encoded flagellin given IM enhanced splenic CD4+ and CD8+ T cell responses to co-expressed vaccine antigen, including memory responses. Boosting either IM or intranasally with Ad vectors expressing Ag85B without flagellin led to durable enhancement of Ag85B-specific antibody and CD4+ and CD8+ T cell responses in both spleen and pulmonary tissues, correlating with significantly improved protection against challenge with pathogenic aerosolized *M*. *tuberculosis*. However, inclusion of flagellin in both DNA prime and Ad booster vaccines induced localized pulmonary inflammation and transient weight loss, with route-dependent effects on vaccine-induced T cell immunity. The latter included marked reductions in levels of mucosal CD4+ and CD8+ T cell responses following IM DNA/IN Ad mucosal prime-boosting, although antibody responses were not diminished. These findings indicate that flagellin has differential and route-dependent adjuvant activity when included as a component of systemic or mucosally-delivered gene-based prime-boost immunization. Clear adjuvant activity for both T and B cell responses was observed when flagellin was included in the DNA priming vaccine, but side effects occurred when given in an Ad boosting vector, particularly via the pulmonary route.

## Introduction

Toll-like receptors (TLRs) are pattern recognition receptors (PRRs) expressed by a variety of cell types, particularly immune cells such as monocytes, macrophages, dendritic cells (DCs), and lymphocytes. TLRs detect pathogen-associated molecular patterns (PAMPs) and their ligation on innate immune cells leads to maturation and activation of these cells, resulting in the initiation of innate immunity. Activation of antigen presenting cells is also required to initiate strong adaptive immune responses [1, 2]. Flagellin, the toll-like receptor 5 ligand (TLR5L), is a highly conserved bacterial protein and the major component of bacterial flagella. Flagellin mediates TLR-5-dependent signal transduction and elicits inflammatory responses in mammalian cells [3, 4], including their production of pro-inflammatory cytokines and upregulation of co-stimulatory molecules on antigen-presenting cells (APCs) [5, 6]. Flagellin is effective at very low concentrations and does not promote potentially deleterious IgE responses. In addition, prior exposure does not appear to impair its adjuvant activity, while antigen sequences can be inserted at the amino or C terminus, or within its hypervariable region, without any loss of activity. Flagellin can also easily be made in large amounts under GMP conditions [7–9]. For these reasons, flagellin is considered to have potential as an adjuvant for vaccines and immunotherapy.

Several studies in animal models have described the adjuvant effects of flagellin, including *Salmonella* FliC, on the induction of host immune responses following parenteral or mucosal immunization, with the majority of these studies focused on antibody responses [3–5, 10–19]. In these studies, flagellin was either mixed with or genetically fused to recombinant protein or peptide vaccines. Its adjuvanticity has also been tested in the setting of a live attenuated bacterial vaccine based on *Salmonella dublin*, in which target antigen was fused to the hypervariable region of flagellin and expressed as chimeric molecules in flagella [12, 20, 21].

There are, however, limited reports of the efficacy of flagellin as an adjuvant in the setting of gene-based vaccines, including strategies designed to induce high-level or sustained immune responses, particularly when administered via different routes. Heterologous prime-boost immunization has been shown to enhance both the magnitude and quality of vaccine-induced immune responses and their protective efficacy in a variety of disease models [22–29]. A common experimental approach has been to prime with DNA vaccines and boost with attenuated viral vectors, each encoding the same vaccine antigens. Despite their capacity to prime immune responses for subsequent boosting, DNA vaccines are often poorly immunogenic when used alone, compared to traditional protein-based immunogens [13, 30, 31].

This model therefore provided the opportunity to test the capacity of flagellin to augment the immunogenicity of DNA vaccine priming for a subsequent viral vector boost. We were also interested in testing the adjuvanticity of flagellin when included in boosting vectors given either by parenteral or mucosal routes. To this end, we have engineered recombinant DNA vaccines and adenovirus vaccine vectors encoding modified *Salmonella typhimurium* flagellin (FliC) along with an immunogenic vaccine antigen, in this case mycobacterial antigen 85B (Ag85B). Ag85B, a fibronectin-binding protein and a major secretory protein in actively replicating *Mycobacterium tuberculosis* (*Mtb*), is a member of the antigen 85 complex family which possess the mycoyltransferase activity required for mycobacterial cell wall formation [32, 33]. In evaluating the adjuvant activity of flagellin in the setting of gene-based prime-boost immunization, we have made several interesting observations. It was clear that vector-driven *S*. *typhimurium* FliC has the capacity to enhance both specific humoral immunity, and also CD4+ and CD8+ T cell responses, when included in the DNA vaccine priming phase of heterologous prime-boost vaccination. Flagellin encoded in DNA vaccines also primed for enhanced vaccine specific immunity following subsequent boosting with viral vectors encoding Ag85B but not flagellin and given either parenterally or mucosally via the intranasal route, in which case both circulating and pulmonary immune responses were enhanced. However, when flagellin was included in both DNA priming and Ad booster vaccines, route-dependent adjuvant effects were apparent, with localized pulmonary inflammation and transient loss of body mass.

## Materials and Methods

### Vaccine vectors

The nucleotide sequence of flagellin (FliC) of Salmonella typhimirium (GenBank Acc.No. EF057754.1) was modified by removal of eukaryotic N-linked glycosylation sites and addition of the murine IL-2 secretion signal to the 5’ prime end. The nucleotide sequence of antigen 85B (Ag85B) of *M*. *tuberculosis* Erdman strain (GenBank Acc.No. X62398) was codon-optimized using the Java codon Optimization tool (http://www.jcat.de). These sequences were manufactured by GenScript (Piscataway, NJ) as synthetic genes and cloned into the pBudCE4.1 plasmid (Invitrogen, Carlsbad, CA), a dual expressing vector, under control of the EF-1α promoter (FliC) using NotI and XhoI restriction enzymes or the CMV promoter (Ag85B) using BamHI and Hind III restriction enzymes. The integrity of resultant pBudCE4.1 constructs were confirmed by restriction digestion and sequencing. Stocks of these constructs were generated using endotoxin-free Megaprep kits (QIAgen, Gaithersburg, MD) and tested for endotoxins by Limulus amebocyte lysate test (Charles River, Wilmington, MA). Recombinant adenovirus vectors encoding flagellin were constructed by cloning the FliC nucleotide sequence into Gateway^®^ pENTR2B entry pAd/CMV/V5-DEST destination vectors (Invitrogen), and recombinant adenovirus type 5 vectors were purified from transfected 293A cells (Life Technologies, Grand Island, NY) by anion exchange chromatography and CsCl density gradient centrifugation. Vectors were tested for presence of flagellin by PCR of viral DNA using flagellin-specific primers. Adenovirus vectors encoding Ag85B, also constructed using Gateway^®^ technology, were previously prepared in this laboratory.

Expression of inserts was tested by Western blotting and biological assay. 293A cells were grown in 6-well plates in DMEM medium (Gibco, Grand Island, NY) containing 2% heat inactivated fetal calf serum (FCS). Cells were transfected with DNA vectors using Lipofectamine 2000 (Invitrogen) according to the manufacturer’s specifications, or transduced with recombinant adenovirus vectors at MOI = 10. Supernatants from DNA-transfected or adenovirus-transduced cells were used to test for expression of flagellin by Western blot using rabbit anti-FliA anti-sera (generously provided by Dr. Eduardo Davila, LSUHSC) at 1:1000 dilution or anti-FliC monoclonal antibody (BioLegend, San Diego, CA, catalog # 629701) at 1:1000 dilution, and for Ag85B using rabbit anti-mouse anti-Ag85 complex polyclonal antibody (BEI Resources, NIAD, NIH: NR-13800) at 1: 5000 dilution. The biological activity of vector-encoded flagellin was tested using THP1-Blue-CD14 cells (InvivoGen, San Diego, CA), human monocyte TLR reporter cells, according to the manufacturer’s specifications. These cells express CD14 and various TLRs including TLR-5, and are stably transfected by a reporter plasmid expressing a secreted embryonic alkaline phosphatase (SEAP) under the control of the NFkB promoter. Cells were grown in 12-well plates in RPMI 1640 medium (Gibco) containing 10% heat inactivated FCS (CM10) and supplemented with 100 μg/mL of zeocin. For assay, cells were incubated with 100 μL of supernatants from DNA-transfected or adenovirus-transduced 293A cells at 37°C 37°C in 5% CO2 for 24 hours. 50 μL of the mixtures were then added to QUANTI-Blue substrate (InvivoGen) in 96-well flat bottom plates in triplicate, incubated at 37°C for 30 minutes, and then read by spectrophotometer at 620 nm.

### Mice and immunizations

Six to eight week old specific pathogen-free female BALB/c mice were purchased from Charles River (Raleigh, NC) and housed in the Animal Care Facility at Louisiana State University Health Sciences Center (LSUHSC). All procedures were approved by the LSUHSC Institutional Animal Care and Use Committee (IACUC). Mice challenged with *M*. *tuberculosis* were housed in a biocontainment level 3 Laboratory (BSL3) operated in accordance with appropriate safety precautions as recommended by the Centers for Disease Control and Prevention and monitored by the LSUHSC Institutional Biosafety Committee. All invasive procedures were performed under anesthesia with a mixture of ketamine HCl (100 mg/kg) and xylazine (10 mg/kg) diluted in PBS.

Vaccine groups for this study are shown in Table 1. For DNA vaccination, mice were immunized twice (3 weeks apart) with 30 μg of plasmid DNA intramuscularly into each tibialis muscle (60 μg total) followed immediately by electropration (IM/EP) with 2 pulses at 150mV using a BTX ECM 830 Electroporator apparatus with caliper electrodes (BTX—Harvard Biosciences, Holliston, Massachusetts). For prime-boosting, mice primed with DNA vaccines were given a booster comprising a cocktail of Ad vaccines. These were suspended in 100 μL PBS and given intramuscularly (5 x 10^8^ PFU total consisting of 2.5 x 10^8^ PFU Ad-85B and 2.5 x 10^8^ PFU Ad-FliC or control Ad-C), or in 20 μL PBS and given intranasally (1x10^8^ PFU total consisting of 5 x 10^7^ PFU Ad-85B and 5 x 10^7^ PFU Ad-FliC or Ad-C). In challenge experiments, mice immunized with BCG (1 x 10^6^ CFU in 100 μL PBS) subcutaneously via footpad at the time of Ad boosting (14 weeks before challenge) served as controls.

**Table 1:** DNA and Prime-Boost Immunizations.

Group Name	Week 0	Week 3	Week 6
	IM/EP	IM/EP	IM or IN
***DNA immunization***			
*DNA-85B-FliC*	pBud-85B-FliC	pBud-85B-FliC	–
*DNA-85B*	pBud-85B	pBud-85B	–
*DNA-FliC*	pBud-FliC	pBud-FliC	–
*DNA-Ctrl*	pBud-Ctrl	pBud-Ctrl	–
***DNA prime—Ad boost immunization***			
*85B-FliC/85B-FliC*	pBud-85B-FliC	pBud-85B-FliC	Ad-85B+Ad-FliC
*85B-FliC/85B*	pBud-85B-FliC	pBud-85B-FliC	Ad-85B+Ad-Ctrl
*85B/85B*	pBud-85B	pBud-85B	Ad-85B+Ad-Ctrl
*FliC/FliC*	pBud-FliC	pBud-FliC	Ad-Ctrl+Ad-FliC
*Ctrl/Ctrl*	pBud-Ctrl	pBud-Ctrl	Ad-Ctrl

DNA vaccines were given intramuscularly followed by electroporation (IM/EP) as described in Materials and Methods, such that each animal received a total of 60 μg of DNA vaccines at weeks 0 and 3. For prime-boost immunization, a total of 5×10^8^ (IM) or 1×10^8^ (IN) PFU of Ad vaccines were given at week 6.

### Ethics Statement

All procedures involving mice were approved by the Louisiana State University Health Sciences Center Institutional Animal Care and Use Committee (IACUC), Approval Number 3010. All invasive procedures were performed under anesthesia with a mixture of KetaThesia (ketamine HCl) and xylazine diluted eight-fold in phosphate-buffered saline. Mice were euthanized by cervical dislocation under anesthesia at the termination of each experiment. Mice were monitored daily throughout these studies for signs of distress, including hunched posture, respiratory distress, or loss of greater than 15% of initial body mass. The experimental plan included early euthanasia of mice displaying any of these characteristics.

### *M*. *tuberculosis* aerosol challenge

Mice were challenged with 50–100 CFU of virulent *M*. *tuberculosis* H37Rv strain (ATCC No. 27294, Rockville, MD) in a certified BSL-3 facility using a GlasCol aerosol infection system (Terre Haute, IN). At week 6 post-challenge, mice were euthanized and lungs and spleens were removed, homogenized, and bacterial loads in these organs enumerated using serial dilutions of homogenate plated on Middlebrook 7H10 agar plates (BD, Sparks, MD), supplemented with OADC (Sigma-Aldrich, St. Louis, MO). The plates were then incubated at 37°C for 2–3 weeks before colonies were counted.

### Peptides

Synthetic H-2^d^-restricted peptide oligomers representing H-2^d^ -restricted CD4+ T cell epitopes in Ag85B, (TFLTSELPQWLSANRAVKPT, HSWEYWGAQLNAMKGDLQ) [34], or a CD8+ T cell epitope, (MPVGGQSSF) [35], were used to stimulate antigen-specific responses in IFN-γ ELISpot assay. All peptides were synthesized by Genscript.

### Isolation of mononuclear cells

Animals were euthanized and spleens were removed, minced with a 3 mL syringe plunger, and passed through a 100 μm nylon mesh cell strainer (BD Falcon, Franklin Lakes, NJ) using RPMI 1640 medium (Gibco) supplemented with 5% heat-inactivated FCS (CM5). RBC’s were lysed using RBC lysis buffer (Sigma-Aldrich) and cells were washed twice with CM5 medium, passed through a 70 μm nylon mesh cell strainer (BD Falcon), and then resuspended in RPMI 1640 medium containing 10% FCS (CM10). Lungs were cut into pieces of less than 0.5 cm in any dimension and incubated for 90 minutes in CM10 supplemented with DNase I at 30 μg/mL and collagenase I at 2 mg/mL (Worthington Biochemical Corp, Lakewood, NJ). Single-cell suspensions were prepared as above.

### IFN-γ ELISpot assay

IFN-γ ELISpot assays for CD4+ and CD8+ T cell responses to peptides were performed and read as described elsewhere [36]. Data are presented as spot-forming cells (SFCs) per million cells ± SEM.

### Intracellular cytokine staining (ICS) assay and tetramer staining

Single cell suspensions at 1–2 x 10^6^ cells per well were stimulated in 96-well round bottom plates (Corning Inc, Corning, NY) with 5 μg/ml of CD4+ and CD8+ T cell peptides at 37°C 5% CO2 for 2 hours, treated with 0.1 μL/well BD GolgiPlug Protein Transport Inhibitor (BD Pharmingen, San Diego, CA), and incubated for an additional 4 hours. Wells containing CM10 medium and PMA/Ionomycin (0.1 mg/mL PMA, 1μg/mL Ionomycin) were used as negative and positive controls respectively. Cells were washed once with FACS buffer (PBS containing 1% FBS), and stained with the fluorescent antibodies CD3e-Pacific Blue, CD4-FITC,CD8-PE-Cy5, and CD44- PE-Cy7 (BD Pharmingen) in FACS buffer for 30 minutes at 4°C. The cells were then washed once with FACS buffer, permeabilized using CytoFix/Cytoperm^™^ Fixation/Permeabilization Kit (BD Biosciences, San Jose, CA) for 20 minutes at 4°C, and then washed once with 1X Premwash buffer and stained for intracellular cytokines with the fluorescent antibodies IFNγ-APC and IL-2-PE (BD Pharmingen). Cells were subsequently washed twice with PBS, fixed in 1% formaldehyde in PBS, and a total of 200,000 events were acquired using a FACS Canto II flow cytometer (Beckman Coulter, Fullerton, California).

For tetramer staining, single cell suspensions of 1–2 x 10^6^ cells per well were stained with the fluorescent antibodies CD3e-Pacific Blue and CD8-FITC (BD Pharmingen) and the PE-labeled Ag85B-specific tetramer, MVPGGQSSF/H-2^d^ (MHC Tetramer Production Facility, Baylor College of Medicine, Houston, Texas) for 45 minutes at room temperature. Cells were then washed twice with PBS, fixed, and 200,000 events were acquired on the FACS Canto II (Beckman Coulter).

ICS and tetramer data were analyzed using FlowJo Software version 8.8.6 (Tree Star, Ashland, Oregon). For ICS analysis, lymphocyte populations were initially identified by forward-scatter and side-scatter profiles. Lymphocytes positive for CD3 were subsequently sorted into CD3+ CD4+ and CD3+ CD8+ T cell populations. These populations were further sorted into CD4+CD44+ and CD8+CD44+ T cell subsets. Cytokine-secretion was measured in these T cell subsets by ICS. Data are presented as numbers of cytokine-producing cells per million CD4+ or CD8+ T cells ± SEM. For tetramer analysis, lymphocytes positive for CD3 were sorted into CD3+ CD8+ T cell populations and were further sorted into tetramer positive cells. Data are shown as percentages of tetramer positive cells within the parent CD3+CD8+ T cell population.

### Antibody-secreting cell (ASC) ELISpot assay

96-well MultiScreen IP plates were coated with 2 μg/mL recombinant Ag85B protein (BEI Resources: NR-14870) in 100 μL PBS overnight at 4°C. Wells coated with 100 μL CM10 served as background controls. Plates were then washed 5 times with PBS and blocked with 200 μL 5% “Blotting Grade Blocker” nonfat dry milk (NFDM, Bio-Rad, Hercules, CA) for 1 hour at room temperature. A total of 2 x 10^5^ cells were seeded per well and incubated at 37°C in 5% CO_2_ for 4–6 hours. Cells were then discarded, and plates were washed 5 times with PBS and incubated with biotinylated goat anti-mouse IgA antibody (IgA-BIOT; Southern Biotech, Birmingham, AL) diluted in PBS containing 1% BSA for 3 hours at room temperature. Plates were subsequently developed by incubation with 100 μL streptavidin-alkaline phosphatase (GE Healthcare, UK Limited, Little Chalfont Buckinghamshire, United Kingdom) diluted in PBS for 1 hour at room temperature and then washed 5 times with PBS,100 μL of BCIP/NBT substrate (Moss Inc, Pasadena, MD) was added and spots were allowed to develop for 20–30 minutes at room temperature. Numbers of antibody-secreting cells (ASCs) were counted using an AID-ELISPOT counter (AID, Strasburg, Germany). Data are presented as spot-forming cells (SFCs) per million cells ± SEM.

### Antibody ELISA

96-well MaxiSorp polystyrene plates (NUNC, Thermo Fisher Scientific, Rochester, NY) were coated with 2 μg/mL recombinant Ag85B (BEI Resources) in 100 μL PBS and incubated at 4°C overnight. Plates were washed 4 times with PBS containing 0.05% Tween 20 (Sigma-Aldrich, wash buffer) and blocked with 200 μL 5% NFDM at 37°C for 2 hours, washed 4 times with wash buffer and serum samples serially-diluted in PBS/1% FCS (ELISA diluent), were added to wells and incubated at 37°C for 1 hour. Wells incubated with ELISA diluent or naïve mouse serum were used as negative controls. Plates were then washed 6 times and biotinylated goat anti-mouse IgG antibody (Southern Biotech, Birmingham, AL) diluted in ELISA diluent was added, and plates were incubated at room temperature and left for 45 minutes. Plates were washed again 6 times and 100 μL streptavidin-alkaline phosphatase (GE Healthcare UK Limited) diluted in ELISA diluent was added at room temperature for 30 minutes. Plates were subsequently washed 8 times and developed with 100 μL pNPP AP substrate (BioFx Laboratories, Owing Mills, MD) at room temperature for 15–25 minutes. Color development was stopped with 50 μL Stopping Solution (BioFx Laboratories) and plates were read at 405 nm on a Synergy HT Multi-Mode Microplate Reader (BioTek). Data are expressed as mean endpoint antibody titers ± SEM.

### Histopathological analysis

Lungs from mice in different immunization groups were collected at days 1 and 10 post-boost, fixed in Z-fix (Anatech Ltd., Battle Creek, Michigan), and embedded in paraffin. Tissue sections were prepared and stained with hematoxylin and eosin (H&E) and were evaluated in the LSUHSC Morphology and Imaging Core in blinded fashion for evidence of bronchitis, peribronchiolitis, perivasculitis, and alveolitis.

### Multiplex assay for cytokines and chemokines

Mouse sera and bronchoalveolar lavage fluid (BALF) collected from mice at day 1 post-boosting with Ad were evaluated for the presence of cytokines and chemokines using a 23-plex assay kit (Bio-Rad, Hercules, California) used according to the manufacturer’s specifications. Analyses were performed on a Luminex 200 machine (Bio-Rad), and data are presented as mean picogram/mL ± SEM.

### Statistical analysis

One-way analysis of variance (ANOVA) followed by Bonferroni’s post-test and two-tailed unpaired student T-test were used to determine statistical significance. A *P* value < 0.05 was considered significant.

## Results

### Expression of Ag85B and flagellin by recombinant DNA and Ad vaccine vectors

**Fig 1:**
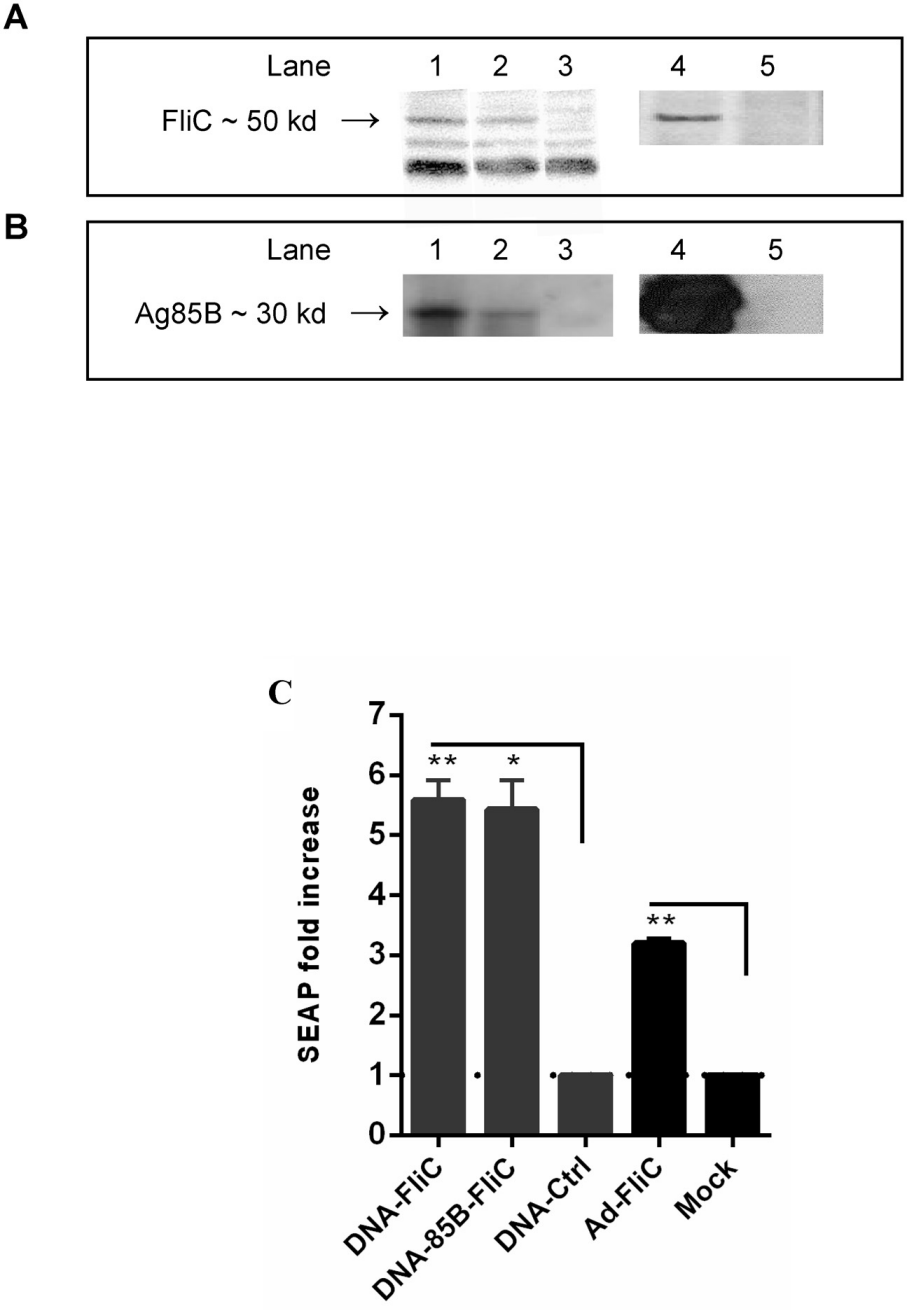
DNA and adenovirus vaccine vectors encoding Ag85B and expression of biologically-active flagellin. (A) Supernatants from DNA-transfected or Ad-infected 293A cells were tested for expression of flagellin by Western blot, lane 1 DNA-FliC, lane 2 DNA-85B-FliC, lane 3 DNA-control, lane 4 Ad-FliC, lane 5 mock. (B) Expression of Ag85B by DNA and Ad vectors was also confirmed by Western blot, lane 1 DNA-85B, 2 DNA-85B-FliC, 3 DNA-FliC, 4 Ad-85B, 5 mock. (C) Biological activity of flagellin was tested by bioassay using THP1-Blue-CD14 cells. SEAP levels were read at 620 nm. Data shown are mean of fold-increase in SEAP ± SEM over respective controls (dotted line), which are supernatants of cells transfected with empty DNA vector (for DNA vaccines), or of mock infected cells (for Ad vaccine) (*p < 0.05; **p < 0.01 vs control). Representative data from one of two independent experiments are shown (n = 3).

Recombinant dual-expression (pBudCE4.1) plasmid vaccine vectors encoding *Salmonella typhimirium* flagellin (FliC) and/or mycobacterial protein Ag85B, and recombinant adenovirus type 5 vaccine vector encoding FliC or Ag85B, were designed and constructed as described above. The expression of flagellin in DNA-FliC, DNA-85B-FliC, and Ad-FliC vaccines (Fig 1A), and of mycobacterial Ag85B in DNA-85B, DNA-85B-FliC, and Ad-85B vaccines (Fig 1B) was confirmed by Western blotting using supernatants from 293A cells transfected with DNA or infected with adenovirus vectors. The biological activity of vector-encoded flagellin was tested using THP1-Blue-CD14 cells, human monocyte TLR reporters stably transfected by a reporter plasmid expressing a secreted embryonic alkaline phosphatase (SEAP) under control of the NFkB promoter. Increases in SEAP expression over empty parent DNA vector (DNA-ctrl) or mock-infected controls were seen respectively, in DNA and Ad vaccine vectors encoding flagellin as read by spectrophotometry (Fig 1C). Preliminary experiments were also conducted using abovementioned supernatants in culture with splenocytes isolated from TLR5 gene knockout mice (B6.129S1-*Tlr5*^*tm1Flv*^ /J, Jackson Labs) or from the recommended control strain (C57Bl6/J, Jackson Labs). It was clear that secretion of the inflammatory factors IL-6, G-CSF, and RANTES, as measured in factor-specific ELISAs, were greatly reduced in 24 hour cultures of DNA-FliC, DNA-85B-FliC, or Ad-FliC supernatant-stimulated cells from the knockout mice compared to control C57Bl6/J splenocytes (data not shown).

### Co-immunization with flagellin encoded in DNA vaccines enhanced antigen-specific CD4+ and CD8+ T cell responses

Initial immunization experiments were designed to evaluate the effects of flagellin encoded in DNA vaccine vectors on CD4+ and CD8+ T cell responses to co-expressed vaccine antigen. As described in Table 1, mice were given two inocula of either DNA-85B-FliC, DNA-85B, or DNA-FliC at an interval of 3 weeks, and both splenic and pulmonary mucosal CD4+ and CD8+ T cell responses were evaluated at 3 weeks after the second immunization.

**Fig 2:**
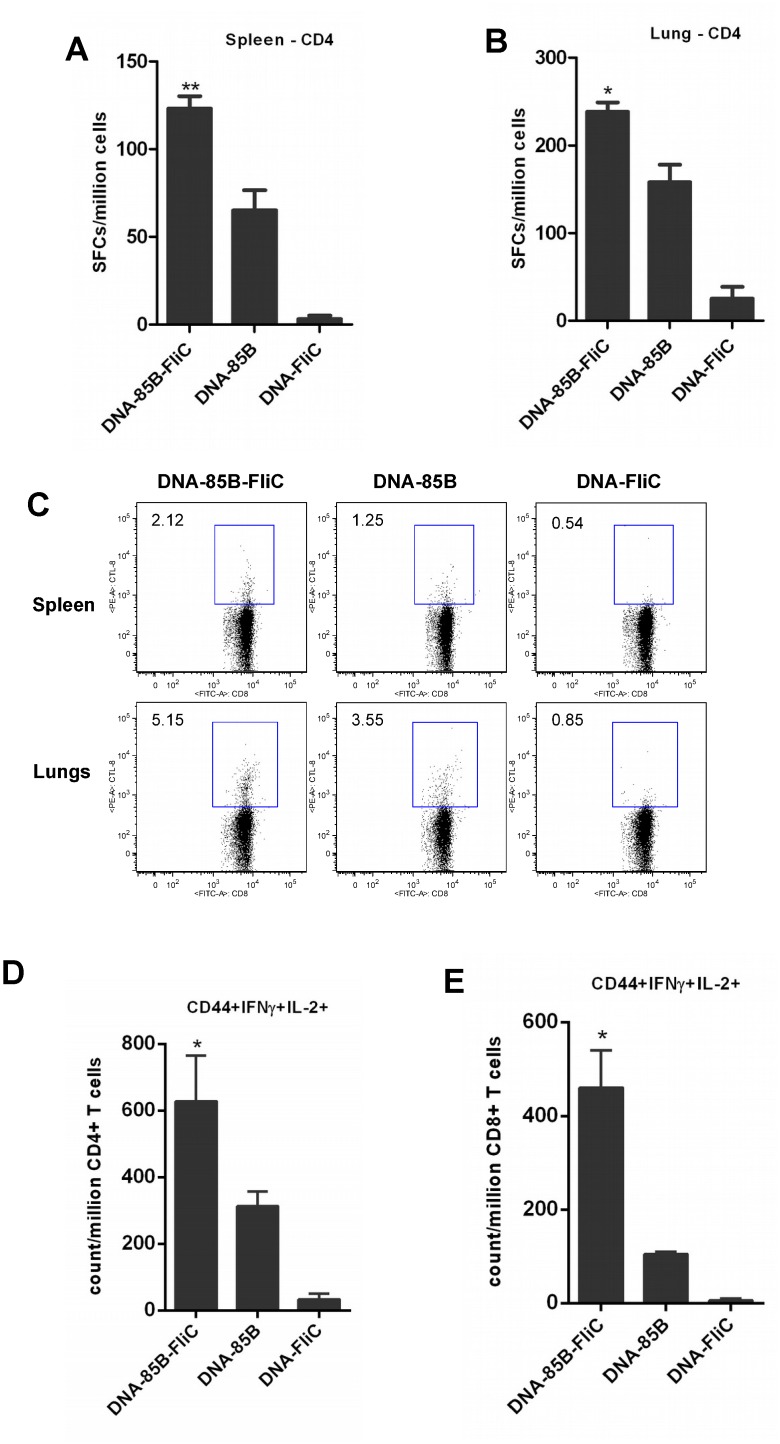
Co-priming with flagellin enhanced Ag85B-specific CD4+ and CD8+ T cell responses following DNA immunization. Mice were immunized twice via the IM route with DNA vaccine encoding Ag85B and/or flagellin as shown in Table 1. At week 3 post-immunization, CD4+ T cell responses in spleen (A) and lung (B) were assayed by IFN-γ ELISpot. Data shown are mean of SFCs ± SEM per group, (* *p* < 0.05; **p < 0.01 vs DNA-85B group). CD8+ T cell responses were assayed in spleen and lungs by tetramer analysis and frequencies of Ag85B-specific tetramer positive cells within the total CD3+CD8+ parent cell population are shown (C). Intracellular cytokine staining for memory CD4+ (D) and CD8+ (E),T cells from spleen was also performed. Data shown are mean counts of Ag85B-specific cytokine-producing CD44+ memory cells per million CD3+CD4+ or CD3+CD8+ parent cells ± SEM (*p < 0.05 vs 85B/85B group). Representative data from one of two independent experiments are shown (n = 5).

Mice co-immunized with Ag85B and flagellin had significant increases in Ag85B-specific CD4+ T cell numbers in both the spleen and the pulmonary mucosa, as evaluated by IFN-γ ELISpot (Fig 2A and 2B). Flagellin co-expression also mediated increased levels of Ag85B-specific splenic and pulmonary mucosal CD8+ T cells measured by tetramer staining (Fig 2C). Further assessment of Ag85B-specific T cell memory responses in the circulation by ICS assay showed that co-priming with flagellin also markedly elevated numbers of vaccine-specific polyfunctional CD44+ memory CD4+ (Fig 2D) and CD8+ (Fig 2E) T cells secreting both IFN-γ and IL-2. These data suggest that co-immunization with DNA-encoded flagellin has the capacity to improve Ag85B-specific CD4+ and CD8+ T cell responses, including the generation of memory responses.

### Co-priming with flagellin enhanced antigen-specific CD4+ and CD8+ T cell responses following heterologous prime-boost immunization

Next, we evaluated the effects of flagellin on antigen-specific CD4+ and CD8+ T cell responses when included in the prime and/or boost component of a heterologous prime-boost immunization. As shown in Table 1, mice were primed twice via the intramuscular (IM) route with DNA vaccines encoding Ag85B and/or flagellin at an interval of three weeks followed by boosting with a cocktail of recombinant Ad vaccine vectors encoding Ag85B and/or flagellin three weeks later.

**Fig 3:**
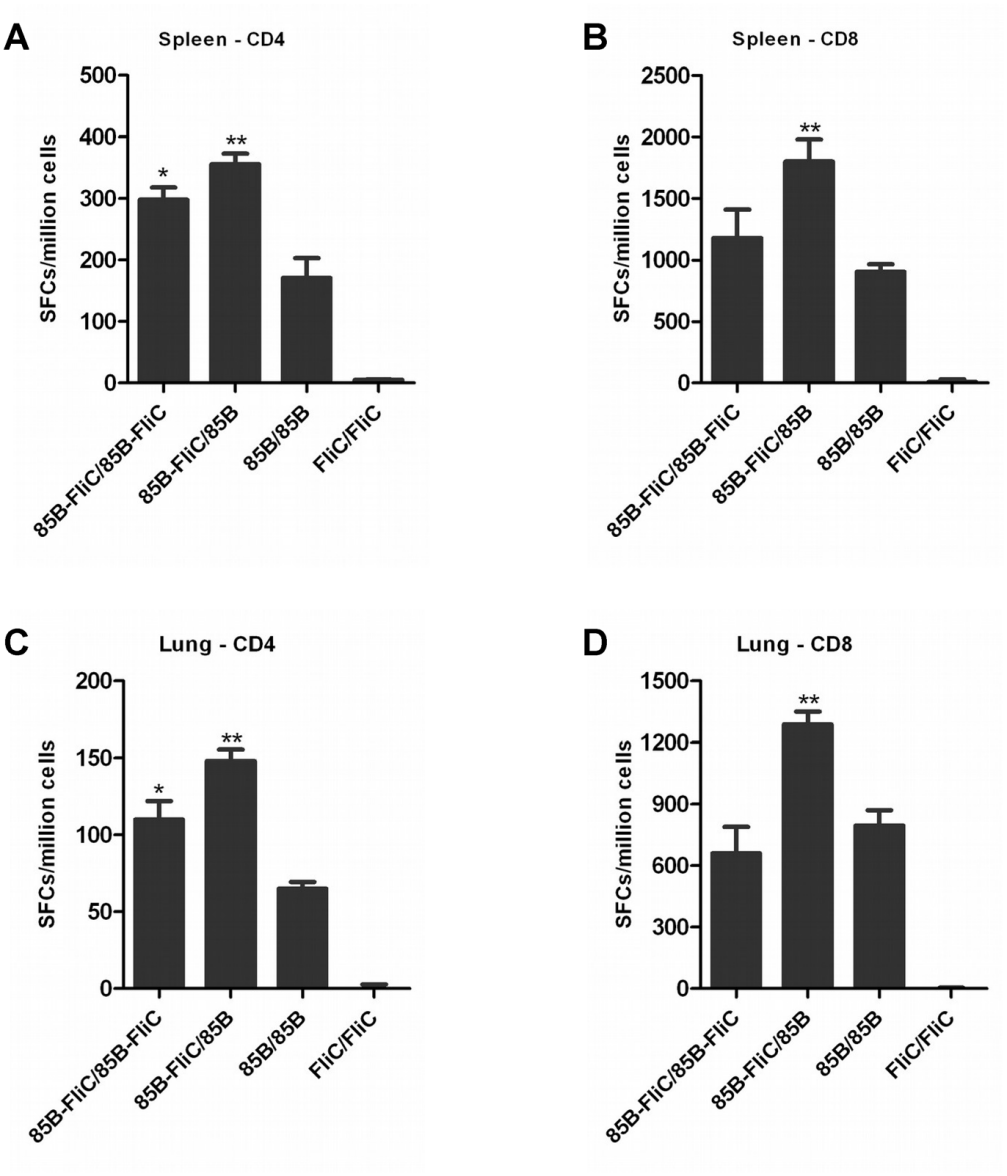
Co-expression of flagellin at the priming phase of immunization increased numbers of splenic and mucosal Ag85B-specific CD4+ and CD8+ T cells. Mice were primed twice via the IM route with DNA vaccines encoding either Ag85B or flagellin, or both, and boosted IM with Ad vaccine vectors encoding Ag85B or flagellin, or with a cocktail of both, three weeks later as shown in Table 1. At week 3 post-boosting, CD4+ T and CD8+ T cell responses in spleen (A&C respectively) and lung (B&D respectively) were assayed by IFN-γ ELISpot. Data shown are mean counts of SFCs ± SEM, (*p < 0.05; **p < 0.01 vs 85B/85B group). Representative data from one of three independent experiments are shown (n = 5).

Splenic and mucosal T cell responses were assessed by IFN-γ ELISpot at week 3 post-boosting. Co-immunization with flagellin enhanced numbers of splenic and mucosal Ag85B-specific IFN-γ producing CD4+ T cells, particularly when it was included only in the priming vaccine (Fig 3A and 3C). Significant increases in Ag85B-specific IFN-γ producing CD8+ T cell numbers were also seen in both spleens (Fig 3B) and in lungs (Fig 3D) when flagellin was included only in the priming vaccine. A similar pattern was seen in spleens and in lungs at 20 weeks post-immunization, i.e. 14 weeks post-boosting (data not shown). These results indicate that flagellin has the capacity to durably enhance antigen-specific CD4+ and CD8+ T cell responses in both spleen and pulmonary mucosa, particularly when included in the DNA vaccine prime component of a heterologous systemic prime-boost immunization regime.

### Co-priming with flagellin potentiated the effects of mucosal boosting in enhancing both pulmonary mucosal and splenic CD4+ and CD8+ T cell responses

The capacity of flagellin to prime for enhanced antigen-specific CD4+ and CD8+ T cell responses following boosting via the intranasal (IN) route was also examined. As detailed in Table 1, DNA vaccine-primed mice were boosted IN with a cocktail of recombinant Ad vaccine vectors encoding Ag85B and/or flagellin. Pulmonary mucosal and splenic antigen-specific T cell responses were measured by IFN-γ ELISpot assay at week 3 post-boosting. Initial studies show both pulmonary and splenic CD4+ and CD8+ T cell responses following DNA/Ad prime-boosting via the IM or IN routes respectively, including a direct comparison between DNA-85B-FliC/Ad-85B prime-boost mice and DNA-85B-Flic-primed mice without Ad boosting (S1 Fig). These data confirm significantly elevated CD4+ and CD8+ T cell responses in the DNA/Ad prime-boost groups.

**Fig 4:**
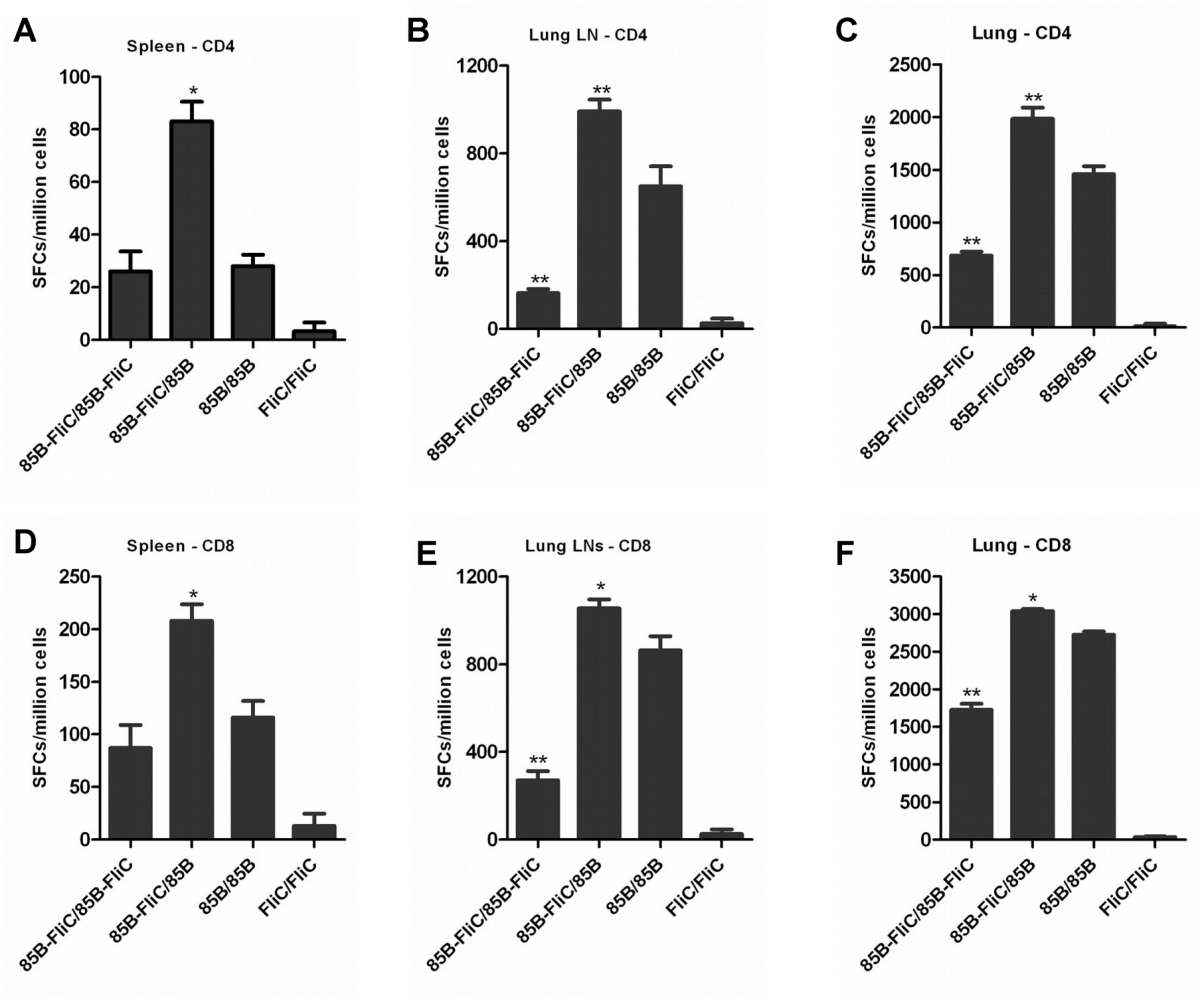
Enhancement of pulmonary mucosal and circulating Ag85B-specific CD4+ and CD8+ T cell responses when flagellin was co-expressed in the priming phase of mucosal prime-boost immunization. Mice were primed twice with DNA vaccine via the IM route and boosted IN with Ad vaccine encoding Ag85B and/or flagellin 3 weeks later as shown in Table 1. At week 3 post-boosting, CD4+ and CD8+ T cell responses in spleen (A&D respectively), lung-associated lymph nodes (B&E respectively), and peripheral lung tissues (C&F respectively),were assayed by IFN-γ ELISpot. Data shown are mean of SFCs ± SEM, (*p < 0.05; **p < 0.01 vs 85B/85B group). Representative data from one of three independent experiments are shown (n = 5).

When included only in the priming vaccine, flagellin significantly enhanced numbers of Ag85B-specific IFN-γ-producing CD4+ and CD8+ T cells in spleen (Fig 4A and 4D), pulmonary lymph nodes (Fig 4B and 4E), and in lung tissues (Fig 4C and 4F), compared to mice immunized with vectors encoding Ag85B alone. These responses persisted for at least 14 week post-boosting (data not shown). Flagellin co-expression in both priming and boosting vectors did not significantly impact Ag85B-specific CD4+ and CD8+ T cell responses measured in the spleen (Fig 4A and 4D), but significantly decreased Ag85B-specific CD4+ and CD8+ T cell responses measured in the lung lymph nodes (Fig 4B and 4E) and in peripheral lung tissues (Fig 4C and 4F) compared to Ag85B-immunized mice. Mucosal CD4+ and CD8+ responses persisted at lower numbers in these mice at week 14 post-boosting (data not shown). It was therefore concluded that co-priming with flagellin enhanced both pulmonary mucosal and splenic Ag85B-specific CD4+ and CD8+ T cell responses induced by heterologous mucosal boosting. Unlike systemic prime-boosting, the inclusion of flagellin in both priming and boosting phases had a negative effect on mucosal T cell responses.

### Flagellin enhanced both circulating and pulmonary mucosal antibody responses following systemic or mucosal prime-boost immunization

**Fig 5:**
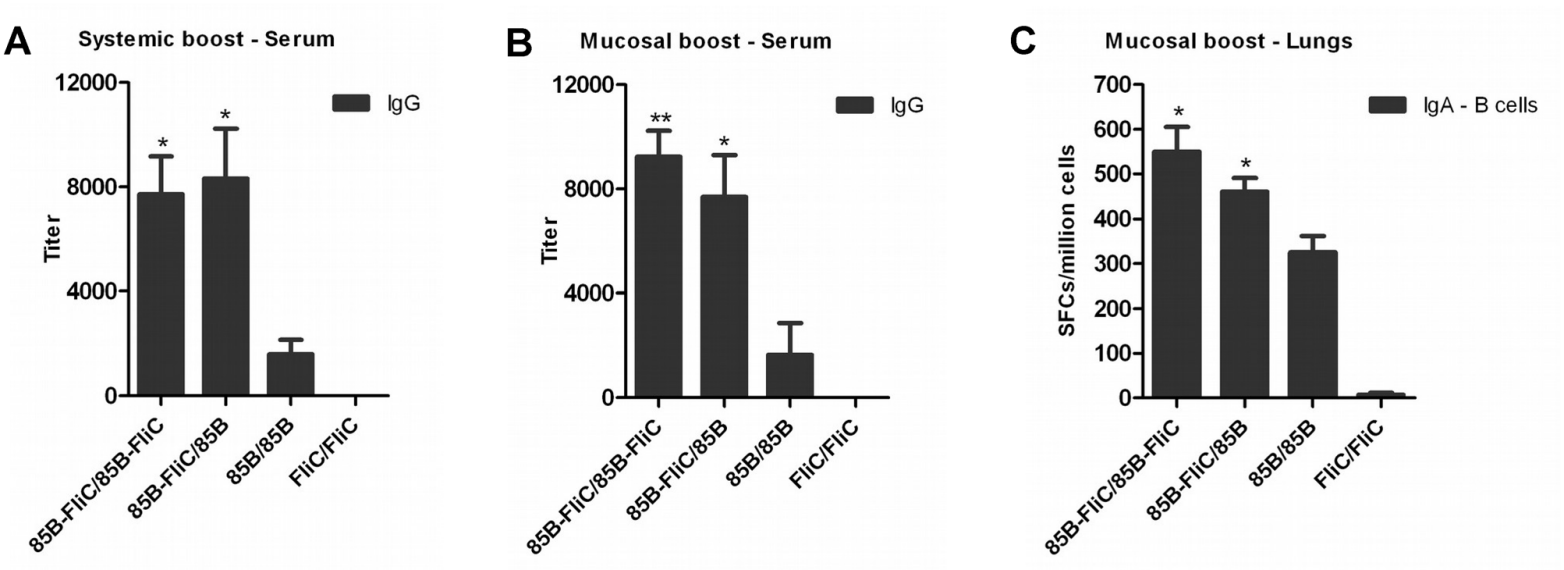
Flagellin enhanced anti-Ag85B antibody responses following either systemic or mucosal prime-boost immunization. Mice were primed twice with DNA vaccine via the IM route and boosted either IM or IN with Ad vaccine three weeks later as shown in Table 1. Sera were collected and tested by ELISA for anti-Ag85B IgG antibodies at week 3 post systemic (A) or mucosal (B) boosting. Data shown are means of endpoint antibody titers ± SEM per group. (C) Lungs were harvested from IN-boosted mice at week 14 post-boosting and tested for mucosal IgA-secreting B cells by ELISpot. Data shown are mean of SFCs ± SEM per group. (*p < 0.05; **p < 0.01 vs 85B/85B group). Representative data from one of two independent experiments are shown (n = 5).

Having found differential effects of flagellin on antigen-specific T cell responses when expressed in different phases of prime-boosting, or when given by different routes, it was of interest to study its effects on antigen-specific antibody responses in this system. DNA-primed mice were boosted by Ad vaccine via either the IM or IN route as described above. Sera were collected and anti-Ag85B IgG responses were estimated by ELISA. Numbers of IgA-secreting B cells in the lungs were measured by ELISpot. At week 3 post-boosting, Ag85B-specific IgG serum antibody levels were significantly increased when flagellin was included in the priming vaccine, or in both priming and boosting phases, following systemic or mucosal prime-boosting (Fig 5A and 5B). These differences persisted at 14 weeks post-boosting (data not shown). Flagellin expression also increased numbers of Ag85B-specific pulmonary IgA-secreting B cells assessed at week 14 post-boosting via the IN route (Fig 5C). These data indicate that flagellin enhanced vaccine-specific circulating and mucosal antibody responses when included in the priming vaccine and, unlike its effects on T cell responses, also increased antibody levels when expressed in both priming and boosting vectors, regardless of the route of boosting.

### Flagellin co-expression in the priming phase improved the protective efficacy of systemic or mucosal prime-boost immunization against *M*. *tuberculosis* infection

**Fig 6:**
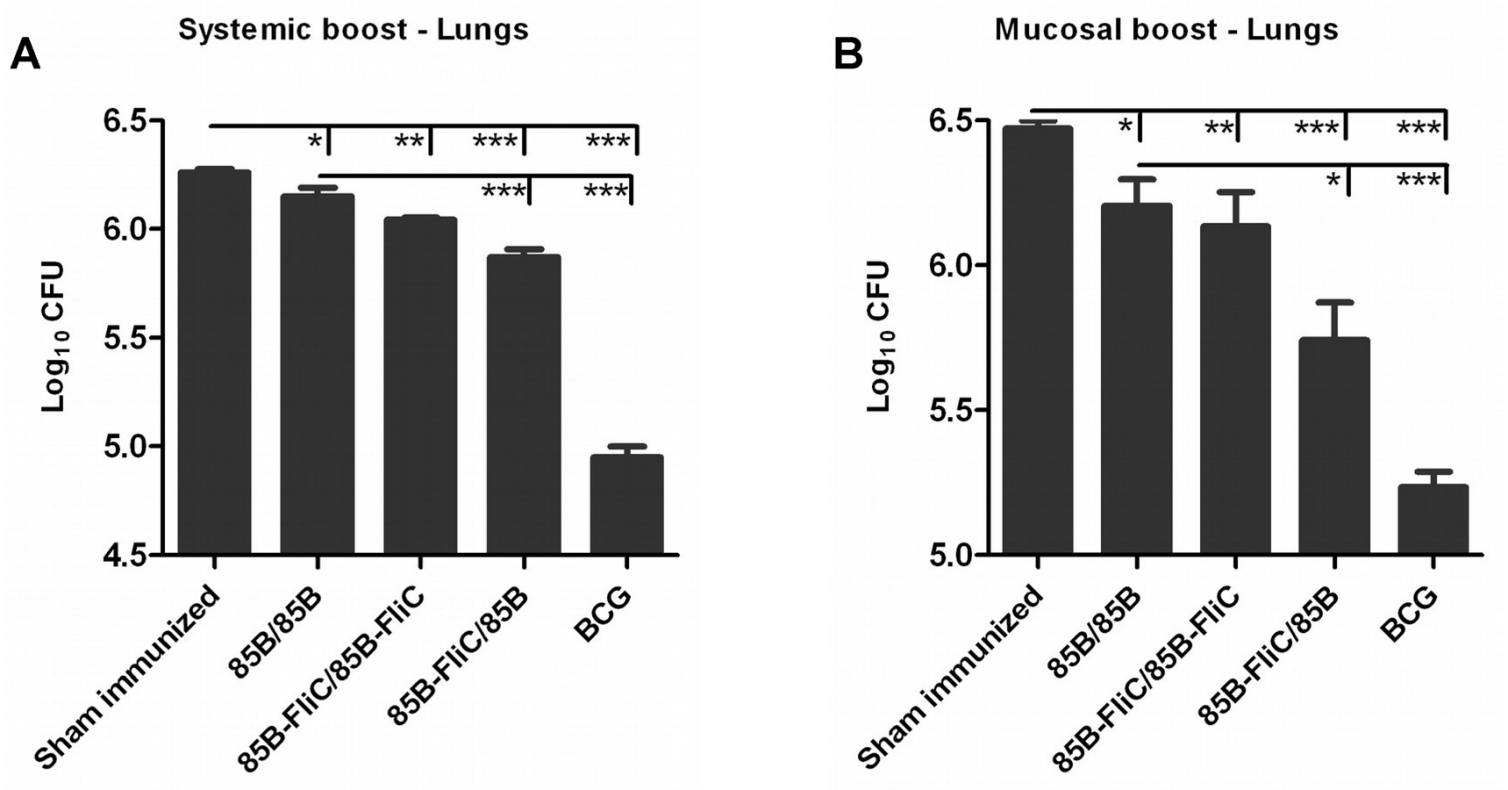
Co-delivery of flagellin improved protective efficacy of vaccination against aerosol challenge with *M*. *tuberculosis*. Mice were primed IM twice with DNA vaccine and boosted either IM or IN with Ad vaccine 3 weeks later or were sham immunized with PBS at these time points as shown in Table 1. Mice given 1×10^6^ CFU of BCG via footpad at 14 weeks prior to challenge, the time point corresponding to Ad boosting, served as controls. At week 14 post-boosting, mice were aerosol challenged with 50–100 CFU of virulent *Mtb* H37Rv strain. At week 6 post-challenge, mice were euthanized and lungs were removed and homogenized for enumeration of bacterial loads in systemic (A) and mucosal (B) prime-boosted mice. Data shown are mean of CFU ± SEM per group for 5 individual mice within each group. (*p < 0.05; **p < 0.01; ***p < 0.001). Representative data from one of two independent experiments are shown (n = 5).

We also used these constructs to examine whether flagellin co-expression improved the protective efficacy of prime-boosting in a murine model of pulmonary TB infection. As detailed in Table 1, DNA-primed mice were boosted with Ad vaccine via IM or IN routes. Sham-immunized (PBS-treated) and BCG-immunized animals were included as negative and positive controls respectively. At week 14 post-boosting, mice were challenged with virulent aerosolized *Mtb* H37Rv strain, and euthanized 6 weeks later for assessment of bacterial loads in the lungs. Inclusion of flagellin in both priming and boosting phases showed only marginal reduction in bacterial growth compared to sham immunized mice, similar to prime-boosting with Ag85B alone (Fig 6A and 6B). However, inclusion of flagellin only in the DNA vaccine prime followed by systemic or mucosal boosting with Ad-encoded Ag85B mediated a highly significant reduction in pulmonary bacterial load (Fig 6A and 6B), particularly following mucosal boosting (7-fold reduction in lungs compared to sham immunized mice). These data indicate that co-delivery of flagellin only in the DNA priming vaccine markedly enhanced protective efficacy in the setting of gene-based heterologous prime-boosting, but that this effect diminished when flagellin was also expressed in the boosting phase. These outcomes correlated with the differential enhancement of Ag85B-specific T cell immunity in this system.

### Morbidity associated with flagellin co-expression in adenovirus boosting vectors

**Fig 7:**
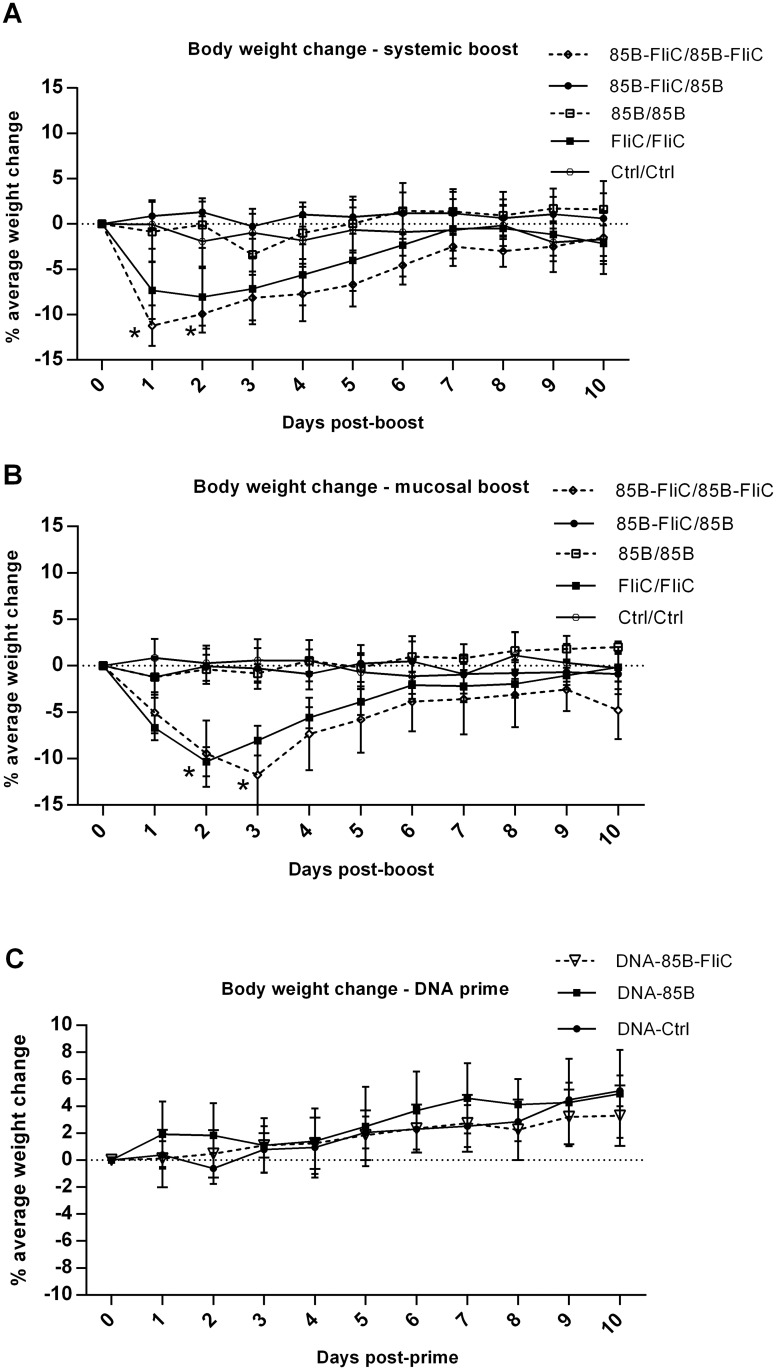
Boosting with Ad-encoded flagellin via intramuscular or intranasal routes caused transient morbidity in mice. Mice were primed twice with DNA vaccine via the IM route, and some groups were boosted either IM or IN with Ad vaccine 3 weeks later as shown in Table 1, Body mass was monitored post-systemic (A) or mucosal (B) boosting with Ad vaccines, or post-DNA priming (C) Data shown represent percentage of average change in body mass ± SEM per group for 5 individual mice within each group (*p < 0.05 vs 85B/85B group). Representative data from one of two independent experiments are shown (n = 5).

**Fig 8:**
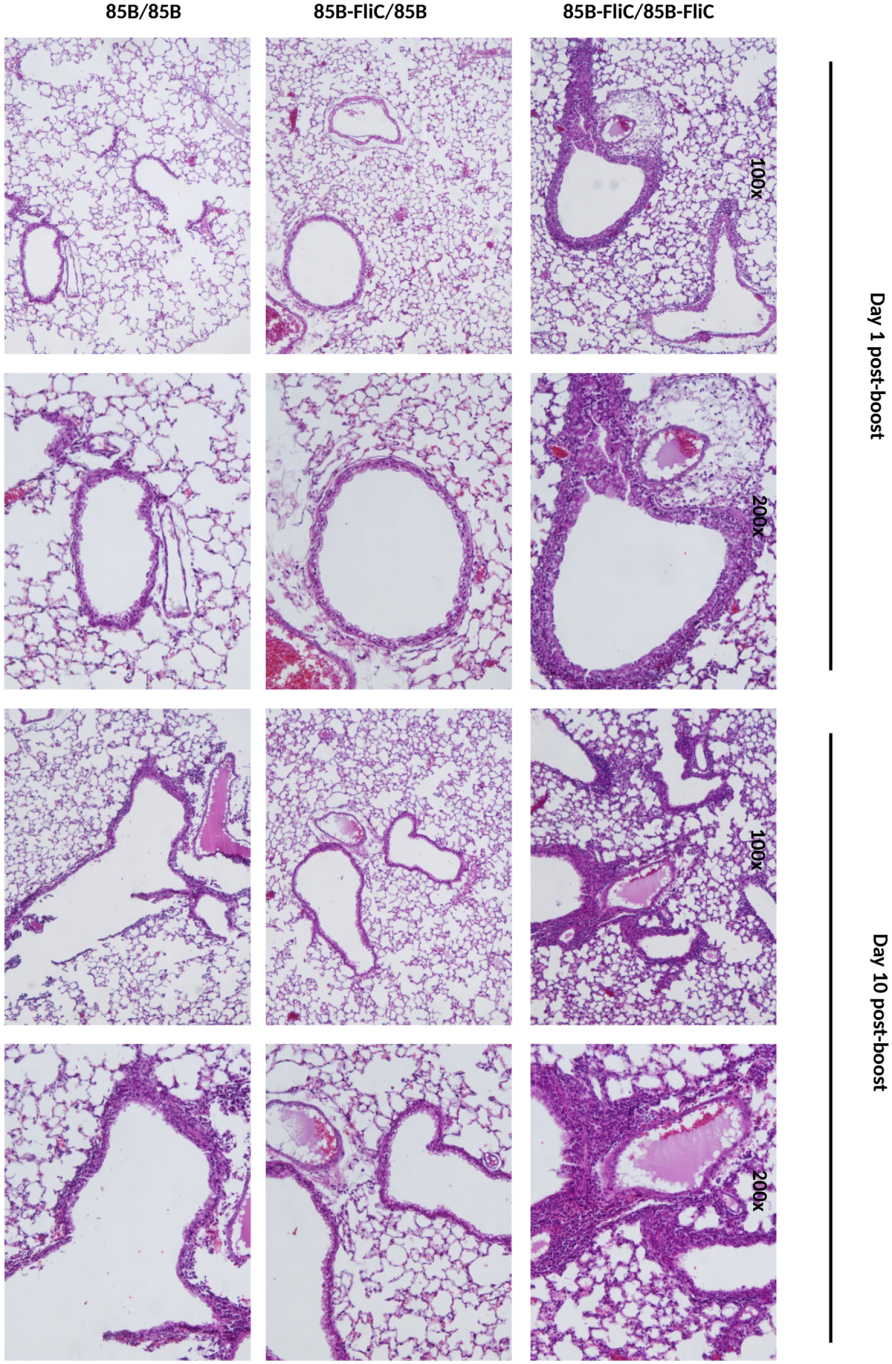
Intranasal delivery of Ad-encoded flagellin caused lung pathology. Mice were primed twice with DNA vaccine via the IM route and boosted IN with Ad vaccine 3 weeks later as shown in Table 1. At days 1 and 10 post-boosting, mice were euthanized, lungs were harvested from different vaccine groups and lung sections were prepared for H&E staining and histopathological evaluation. Representative lung sections from mice in different vaccine groups are shown at 100x and 200x magnification. Representative data from one of two independent experiments are shown (n = 3).

We evaluated the safety of constructs encoding flagellin, particularly in the light of our findings showing diminished mucosal T cell immunity and reduced protective efficacy in mice boosted with Ad vectors encoding this molecule. Several features, including body mass, were evaluated daily following either DNA priming or DNA/Ad prime-boosting. As shown in Fig 7, either systemic (Fig 7A) or mucosal (Fig 7B) boosting of DNA-primed mice with Ad expressing flagellin led to transient but significant loss of body mass, most noticeably on days 1–3, and this was accompanied by ruffled fur and reduced activity. None of these effects were seen in mice given flagellin only in the priming vaccine, similar to mice given vectors encoding Ag85B but not flagellin, or in mice given ‘empty’ control DNA and Ad vaccines (Fig 7A and 7B) or in control mice that were not immunized (data not shown). Mice given only DNA vaccines encoding Ag85B with or without flagellin, or ‘empty’ control DNA vaccine, had no loss of body mass (Fig 7C) or other apparent morbidity. Histopathological analyses of lung tissues at days 1 and 10 following IN boosting with Ad vaccine were also performed. Representative lung sections stained with H&E showed that mice given flagellin in both DNA prime and Ad booster vaccines demonstrated characteristic features of lung damage, including peribronchial, perialveolar, and perivascular infiltration of inflammatory cells and edema at day 1 post-boosting (Fig 8). Peribronchial and perivascular inflammation persisted at day 10 post-boosting, with discrete patches of fibrosis (Fig 8). In contrast, mice given flagellin in the DNA priming but not in the Ad booster vaccine, or those given DNA and Ad vector-encoded Ag85B without flagellin, had minimal evidence of inflammation at day 1 post-boosting, with relatively mild peribronchial and perivascular inflammation seen at day 10 (Fig 8). Thus, while co-priming with DNA vaccine-encoded flagellin had little or no apparent side-effects, there was significant loss of body mass, albeit transient, following boosting with Ad vectors encoding this factor, along with pulmonary inflammation.

### Boosting with Ad-encoded flagellin increased inflammatory responses

**Fig 9:**
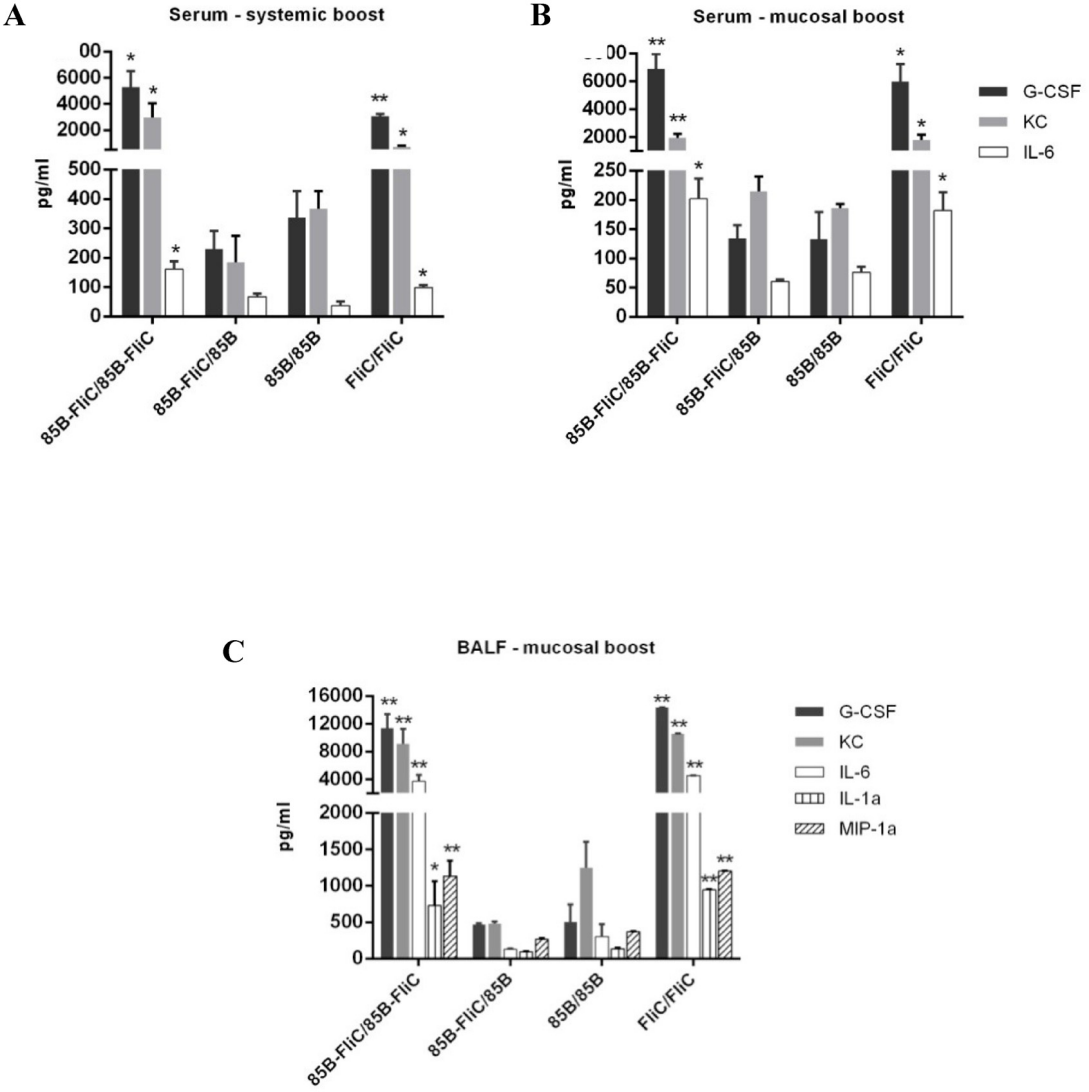
Ad-encoded flagellin increased levels of inflammatory factors in the circulation and pulmonary tissues. Mice were primed twice with DNA vaccine via the IM route and boosted either IM or IN with Ad vaccine 3 weeks later as shown in Table 1. Levels of inflammatory cytokines and chemokines in serum samples following systemic (A) or mucosal (B) boosting, and in BALF (C)post-mucosal boosting were assayed by multiplex as described in Materials and Methods. Samples were collected from mice at 24 hours post-boosting. Cytokine concentrations are depicted as means ± SEM in each group (*p < 0.05; **p < 0.01 vs 85B/85B group). Representative data from one of two independent experiments are shown (n = 4).

Finally, given these findings, we evaluated flagellin-induced inflammatory responses in the vaccinated animals. Mice were immunized as detailed in Table 1 and levels of pro-inflammatory cytokines and chemokines in sera and in BALF collected at day 1 post-boosting with Ad vaccine were measured by multiplex assay. Mice given flagellin in both priming and boosting vaccines had significantly elevated levels of KC, IL-6, and G-CSF in sera at 24h post-systemic or mucosal boosting compared to those immunized with Ag85B alone (Fig 9A and 9B). These inflammatory responses had diminished by day 3 and were no longer apparent at day 10 (data not shown). No upregulation in the levels of these or other inflammatory factors was seen at any of these time points when flagellin was given only in the DNA prime vaccine. Similar findings were recorded in BALF, with levels of IL-1α and MIP-1α also elevated following IN boosting with Ad-encoded flagellin (Fig 9C). These data indicate that acute elevation of inflammatory factors in serum and lung lavage fluids preceded the transient loss of body mass, pulmonary inflammation and reduced T cell responses that were observed only when flagellin was encoded in Ad vaccine boosting vectors.

## Discussion

In this study, several novel observations were made concerning the adjuvant activity of flagellin in the setting of gene-based prime-boost immunization. It was clear that vector-driven *Salmonella typhimurium* FliC has the capacity to durably enhance both specific humoral immunity and CD4+ and CD8+ T cell responses when included in the DNA priming phase of heterologous prime-boost immunization. Thus, flagellin encoded in DNA vaccines primed for enhanced vaccine-specific immunity following subsequent boosting with recombinant adenovirus vectors given either parenterally or mucosally via the intranasal route, in which case both circulating and pulmonary immune responses were enhanced. However, there were clear indications of a route-dependent mode of action when flagellin was included in both DNA priming and Ad boosting phases of vaccination, with marked reductions in mucosal T cell responses.

The capacity of DNA vector-encoded flagellin to enhance antigen-specific CD4+ T cell responses is in accord with previous protein-based studies where flagellin was physically linked or admixed to the vaccine antigen [10, 37, 38]. The ability of flagellin to enhance CD8+ T cells responses is more controversial. Previous studies have shown no significant improvement of CD8+ T cell responses due to flagellin [5, 39], while others reported a positive effect of flagellin on CD8+ T cell immunity [4, 10–12]. In the present study, co-priming with flagellin clearly enhanced vaccine-specific CD8+ T cell numbers, particularly when included in the priming phase of systemic prime-boost immunization. In preliminary studies, increases in percentages of cytotoxic CD8+ T cells expressing the CD107a degranulation marker were also found when flagellin was included in the priming vector (our unpublished data). We also observed that co-priming with flagellin significantly influenced the generation of polyfunctional CD4+ and CD8+ T cells capable of the concomitant production of multiple Th-1 cytokines including IFN-γ, TNF-α and IL-2. These cell populations appear, in some studies, to correlate with improved protective efficacy in a variety of disease models of intracellular infection [24, 40, 41].

In systemic prime-boosting, inclusion of flagellin in both priming and boosting vaccines led to enhancement of the magnitude of vaccine-induced CD4+ T cell responses, although these increases were not as strong as when flagellin was included only in the priming vaccine. However, inclusion of flagellin in both priming and boosting phases of mucosal prime-boost immunization did not appear to enhance CD4+ and CD8+ T cell responses in the circulation and, unexpectedly, led to significant reductions in the magnitude of these responses in the pulmonary mucosa. These novel findings highlight differential and route-dependent adjuvant effects of flagellin when included in vaccine vectors delivered by different routes. The reason for the unexpected inhibitory effects of flagellin on antigen-specific T cell responses following intranasal delivery of Ad vectors is not immediately clear. A possible explanation could be the generation of strong pro-inflammatory responses in the lungs that could negatively affect the expression of T cell responses against the vaccine antigen. Studies are underway to clarify this issue.

The capacity of flagellin to induce antibody responses has been extensively studied. This factor was shown to induce protective antibodies, including secretory IgA, following parenteral or intranasal immunization that were not dependent on tandem expression with the vaccine antigen [3, 10–12, 14–17, 19]. Here, in contrast to its differential and route-dependent adjuvant effects on vaccine-induced T cell responses, flagellin enhanced the induction of antibody responses regardless of the phase of vaccine expression or the route of boosting. In particular, mucosal boosting with Ad-encoded flagellin, which had shown inhibitory effects on CD4+ and CD8+ T cell responses, clearly enhanced IgG and IgA antibody responses to vaccine antigen, extending evidence for the adjuvant activity of flagellin to include its delivery in gene-based vaccines.

Mycobacterial Ag85B, and other members of the Ag85 complex, are highly immunogenic proteins that are currently being tested in several pre-clinical and clinical trials of novel candidate TB vaccines [42, 43]. In this study, the inclusion of the gene encoding Ag85B in our recombinant vaccines allowed us to evaluate of the efficacy of vaccine-induced immunity in protecting against aerosol challenge with *Mtb* in a murine model of acute pulmonary TB infection. Co-priming with Ag85B and flagellin in DNA vaccines followed by boosting with Ad-85B without encoded flagellin significantly reduced bacterial burdens in lungs and in spleens post-challenge, particularly after mucosal prime-boost immunization. This was associated with elevated immune responses against this protein, particularly Ag85B-specific CD4+ T cell responses, including polyfunctional subsets expressing IFN-γ and other Th-1 cytokines that may be important for protection against TB [44, 45].

Flagellin mediated increases in the magnitude of vaccine-induced CD4+ and CD8+ T cell responses in the circulation and in lung tissues, and also increased memory CD4+ and CD8+ T cell populations in the circulation, when given only in the priming DNA vaccine. Generation of immune memory is a hallmark of successful immunization [46, 47], and these responses were further enhanced by boosting with Ad vectors encoding the same vaccine antigen. Flagellin has been shown to promote marked increases in T and B lymphocyte recruitment to draining lymph nodes, increasing the likelihood of these cells encountering their cognate antigen [48], and can also directly stimulate CD4+ and CD8+ T cells [49].

Flagellin given in protein form has been reported in both pre-clinical and clinical trials to be a safe and non-toxic adjuvant [8, 9, 50, 51]. However, high doses were found to cause systemic inflammation and transient liver injury [52]. Here, inclusion of flagellin in an Ad boosting vaccine given via either systemic or intranasal routes caused a transient loss of body mass. Intranasal delivery of Ad-encoded flagellin also caused peribronchitis, perivasculitis, and alveolitis by 24h post-delivery, persisting at day 10. These side effects were accompanied by acute induction of high levels of KC, IL-6, and G-CSF in sera and lung fluids, while MIP-1α and IL-1α were also upregulated in lung fluids from mice intranasally boosted with Ad vectors encoding flagellin. Each of these factors exerts multiple pro-inflammatory effects [53]. In particular, IL-6, G-CSF and MIP-1α stimulate granulocyte production in bone marrow and the recruitment and activation of these cells [53, 54]. KC induces chemotaxis in inflammatory granulocytes and is believed to play a role in the pathogenesis of bronchiolitis [53, 55], while IL-1α has been shown to increase vascular permeability [53, 56]. Our findings are in agreement with previous studies showing that flagellin increases inflammatory responses in mouse lung and human tracheal epithelium [57–59]. This might be attributed to overstimulation of innate immunity via TLR5 signaling [60, 61]. However, inclusion of flagellin in the DNA priming vaccine either alone, or when followed by boosting with Ad encoding only vaccine antigen, did not cause weight loss or any other apparent morbidity. Differences in the kinetics and magnitude of inflammatory responses that are induced following immunization with DNA or Ad vaccines may be attributed to the initial immunization dose, to vector efficiency in terms of their capacity to transfect or transduce cells, or to the amount or duration of vaccine antigen that is expressed [62, 63].

In conclusion, flagellin has differential and route-dependent adjuvant activity when included as a component of systemic or mucosal gene-based prime-boost immunization strategies. Adjuvant activity for both T and B cell responses, with no apparent toxic side effects, was seen when it was delivered in DNA vaccines, in which case it could clearly prime for enhanced protective immune responses upon boosting with heterologous vectors encoding the same vaccine antigen. Studies are underway to further clarify mechanisms underlying its route-dependent effects when delivered in recombinant adenovirus vectors, particularly via mucosal routes.

## Supporting Information

S1 Fig Adenovirus boosting enhanced antigen-specific CD4+ and CD8+ T cell responses primed by DNA immunization. Mice were primed twice via the IM route with DNA vaccines encoding Ag85B or co-expressing Ag85B and flagellin, or with ‘empty’ control DNA vaccine. At 3 wk after the second DNA priming dose, groups of these mice were boosted either IM or IN with Ad vaccine vectors encoding Ag85B (Ag85B-FliC/85B and Ag85B/Ag85B groups), with control Ad vaccine (Ctrl/Ctrl groups), or were not boosted with Ad vaccines (DNA-85B-FliC, DNA-85B, and DNA-Ctrl groups). At week 3 after either Ad boosting, or the second IM DNA priming dose in the groups that were not Ad-boosted, CD4+ T and CD8+ T cell responses in spleen (A and B) and lung tissues (C and D) were assayed by IFN-γ ELISpot. Data shown are mean counts of SFCs ± SEM, (*p < 0.05; **p < 0.01). Representative data from one of two independent experiments are shown (n = 5).
(TIF)

## References

[1] Kaisho T, Akira S. Toll-like receptors as adjuvant receptors. Biochim Biophys Acta. 2002;1589(1):1–13. Epub 2002/03/23. 11909637 10.1016/s0167-4889(01)00182-3

[2] van Duin D, Medzhitov R, Shaw AC. Triggering TLR signaling in vaccination. Trends Immunol. 2006;27(1):49–55. Epub 2005/11/29. 16310411 10.1016/j.it.2005.11.005

[3] Skountzou I, Martin MD, Wang B, Ye L, Koutsonanos D, Weldon W, et al Salmonella flagellins are potent adjuvants for intranasally administered whole inactivated influenza vaccine. Vaccine. 2009;28(24):4103–12. Epub 2009/08/06. 10.1016/j.vaccine.2009.07.058 19654062 PMC3187848

[4] Nguyen CT, Hong SH, Sin JI, Vu HV, Jeong K, Cho KO, et al Flagellin enhances tumor-specific CD8(+) T cell immune responses through TLR5 stimulation in a therapeutic cancer vaccine model. Vaccine. 2013;31(37):3879–87. Epub 2013/07/09. 10.1016/j.vaccine.2013.06.054 23831323

[5] Didierlaurent A, Ferrero I, Otten LA, Dubois B, Reinhardt M, Carlsen H, et al Flagellin promotes myeloid differentiation factor 88-dependent development of Th2-type response. J Immunol. 2004;172(11):6922–30. Epub 2004/05/22. 15153511 10.4049/jimmunol.172.11.6922

[6] Ciacci-Woolwine F, Blomfield IC, Richardson SH, Mizel SB. Salmonella flagellin induces tumor necrosis factor alpha in a human promonocytic cell line. Infect Immun. 1998;66(3):1127–34. Epub 1998/03/06. 9488405 10.1128/iai.66.3.1127-1134.1998 PMC108025

[7] Ben-Yedidia T, Arnon R. Effect of pre-existing carrier immunity on the efficacy of synthetic influenza vaccine. Immunol Lett. 1998;64(1):9–15. Epub 1998/12/29. 9865596 10.1016/s0165-2478(98)00073-x

[8] Honko AN, Mizel SB. Effects of flagellin on innate and adaptive immunity. Immunologic research. 2005;33(1):83–101. Epub 2005/08/27. 16120974 10.1385/IR:33:1:083

[9] Mizel SB, Bates JT. Flagellin as an adjuvant: cellular mechanisms and potential. J Immunol. 2010;185(10):5677–82. Epub 2010/11/05. 10.4049/jimmunol.1002156 21048152 PMC3756556

[10] Cuadros C, Lopez-Hernandez FJ, Dominguez AL, McClelland M, Lustgarten J. Flagellin fusion proteins as adjuvants or vaccines induce specific immune responses. Infect Immun. 2004;72(5):2810–6. Epub 2004/04/23. 15102791 10.1128/IAI.72.5.2810-2816.2004 PMC387897

[11] Huleatt JW, Jacobs AR, Tang J, Desai P, Kopp EB, Huang Y, et al Vaccination with recombinant fusion proteins incorporating Toll-like receptor ligands induces rapid cellular and humoral immunity. Vaccine. 2007;25(4):763–75. Epub 2006/09/14. 16968658 10.1016/j.vaccine.2006.08.013

[12] Braga CJ, Massis LM, Sbrogio-Almeida ME, Alencar BC, Bargieri DY, Boscardin SB, et al CD8+ T cell adjuvant effects of Salmonella FliCd flagellin in live vaccine vectors or as purified protein. Vaccine. 2010;28(5):1373–82. Epub 2009/11/26. 10.1016/j.vaccine.2009.11.003 19932669

[13] Shi W, Li YH, Liu F, Yang JY, Zhou DH, Chen YQ, et al Flagellin enhances saliva IgA response and protection of anti-caries DNA vaccine. Journal of dental research. 2012;91(3):249–54. Epub 2011/10/27. 10.1177/0022034511424283 22027714

[14] Wang BZ, Quan FS, Kang SM, Bozja J, Skountzou I, Compans RW. Incorporation of membrane-anchored flagellin into influenza virus-like particles enhances the breadth of immune responses. J Virol. 2008;82(23):11813–23. Epub 2008/09/13. 10.1128/JVI.01076-08 18786995 PMC2583664

[15] Ben-Yedidia T, Tarrab-Hazdai R, Schechtman D, Arnon R. Intranasal administration of synthetic recombinant peptide-based vaccine protects mice from infection by Schistosoma mansoni. Infect Immun. 1999;67(9):4360–6. Epub 1999/08/24. 10456875 10.1128/iai.67.9.4360-4366.1999 PMC96753

[16] Strindelius L, Filler M, Sjoholm I. Mucosal immunization with purified flagellin from Salmonella induces systemic and mucosal immune responses in C3H/HeJ mice. Vaccine. 2004;22(27–28):3797–808. Epub 2004/08/19. 15315861 10.1016/j.vaccine.2003.12.035

[17] Weimer ET, Ervin SE, Wozniak DJ, Mizel SB. Immunization of young African green monkeys with OprF epitope 8-OprI-type A- and B-flagellin fusion proteins promotes the production of protective antibodies against nonmucoid Pseudomonas aeruginosa. Vaccine. 2009;27(48):6762–9. Epub 2009/09/12. 10.1016/j.vaccine.2009.08.080 19744586

[18] Huleatt JW, Nakaar V, Desai P, Huang Y, Hewitt D, Jacobs A, et al Potent immunogenicity and efficacy of a universal influenza vaccine candidate comprising a recombinant fusion protein linking influenza M2e to the TLR5 ligand flagellin. Vaccine. 2008;26(2):201–14. Epub 2007/12/08. 18063235 10.1016/j.vaccine.2007.10.062

[19] Qian F, Guo A, Li M, Liu W, Pan Z, Jiang L, et al Salmonella flagellin is a potent carrier-adjuvant for peptide conjugate to induce peptide-specific antibody response in mice. Vaccine. 2015;S0264-410X(15):00292–3. Epub 2015/03/15. 10.1016/j.vaccine.2015.03.006 25765964

[20] Newton SM, Joys TM, Anderson SA, Kennedy RC, Hovi ME, Stocker BA. Expression and immunogenicity of an 18-residue epitope of HIV1 gp41 inserted in the flagellar protein of a Salmonella live vaccine. Res Microbiol. 1995;146(3):203–16. Epub 1995/03/01. 7569315 10.1016/0923-2508(96)80276-2

[21] Verma NK, Ziegler HK, Stocker BA, Schoolnik GK. Induction of a cellular immune response to a defined T-cell epitope as an insert in the flagellin of a live vaccine strain of Salmonella. Vaccine. 1995;13(3):235–44. Epub 1995/02/01. 7543230 10.1016/0264-410x(95)93308-v

[22] Skinner MA, Ramsay AJ, Buchan GS, Keen DL, Ranasinghe C, Slobbe L, et al A DNA prime-live vaccine boost strategy in mice can augment IFN-gamma responses to mycobacterial antigens but does not increase the protective efficacy of two attenuated strains of Mycobacterium bovis against bovine tuberculosis. Immunology. 2003;108(4):548–55. Epub 2003/04/02. 12667217 10.1046/j.1365-2567.2003.01589.x PMC1782916

[23] Ramsay AJ, Leong KH, Ramshaw IA. DNA vaccination against virus infection and enhancement of antiviral immunity following consecutive immunization with DNA and viral vectors. Immunol Cell Biol. 1997;75(4):382–8. Epub 1997/08/01. 9315482 10.1038/icb.1997.60

[24] Ramshaw IA, Ramsay AJ. The prime-boost strategy: exciting prospects for improved vaccination. Immunol Today. 2000;21(4):163–5. Epub 2000/03/31. 10740236 10.1016/s0167-5699(00)01612-1

[25] Woodland DL. Jump-starting the immune system: prime-boosting comes of age. Trends Immunol. 2004;25(2):98–104. Epub 2004/04/23. 15102369 10.1016/j.it.2003.11.009

[26] Estcourt MJ, Ramsay AJ, Brooks A, Thomson SA, Medveckzy CJ, Ramshaw IA. Prime-boost immunization generates a high frequency, high-avidity CD8(+) cytotoxic T lymphocyte population. International Immunology. 2002;14(1):31–7. 11751749 10.1093/intimm/14.1.31

[27] Schneider J, Gilbert SC, Blanchard TJ, Hanke T, Robson KJ, Hannan CM, et al Enhanced immunogenicity for CD8+ T cell induction and complete protective efficacy of malaria DNA vaccination by boosting with modified vaccinia virus Ankara. Nat Med. 1998;4(4):397–402. Epub 1998/04/18. 9546783 10.1038/nm0498-397

[28] Goonetilleke NP, McShane H, Hannan CM, Anderson RJ, Brookes RH, Hill AV. Enhanced immunogenicity and protective efficacy against Mycobacterium tuberculosis of bacille Calmette-Guerin vaccine using mucosal administration and boosting with a recombinant modified vaccinia virus Ankara. J Immunol. 2003;171(3):1602–9. Epub 2003/07/23. 12874255 10.4049/jimmunol.171.3.1602

[29] Rigato PO, de Alencar BC, de Vasconcelos JR, Dominguez MR, Araujo AF, Machado AV, et al Heterologous plasmid DNA prime-recombinant human adenovirus 5 boost vaccination generates a stable pool of protective long-lived CD8+ T effector memory cells specific for a human parasite, Trypanosoma cruzi. Infect Immun. 2011;79(5):2120–30. Epub 2011/03/02. 10.1128/IAI.01190-10 21357719 PMC3088135

[30] Dalmia N, Ramsay AJ. Prime-boost approaches to tuberculosis vaccine development. Expert Rev Vaccines. 2012;11(10):1221–33. Epub 2012/11/28. 10.1586/erv.12.94 23176655 PMC3572762

[31] Saade F, Petrovsky N. Technologies for enhanced efficacy of DNA vaccines. Expert Rev Vaccines. 2012;11(2):189–209. Epub 2012/02/09. 10.1586/erv.11.188 22309668 PMC3293989

[32] Belisle JT, Vissa VD, Sievert T, Takayama K, Brennan PJ, Besra GS. Role of the major antigen of Mycobacterium tuberculosis in cell wall biogenesis. Science. 1997;276(5317):1420–2. Epub 1997/05/30. 9162010 10.1126/science.276.5317.1420

[33] D'Souza S, Romano M, Korf J, Wang XM, Adnet PY, Huygen K. Partial reconstitution of the CD4+-T-cell compartment in CD4 gene knockout mice restores responses to tuberculosis DNA vaccines. Infect Immun. 2006;74(5):2751–9. Epub 2006/04/20. 16622212 10.1128/IAI.74.5.2751-2759.2006 PMC1459720

[34] D'Souza S, Rosseels V, Romano M, Tanghe A, Denis O, Jurion F, et al Mapping of murine Th1 helper T-Cell epitopes of mycolyl transferases Ag85A, Ag85B, and Ag85C from Mycobacterium tuberculosis. Infect Immun. 2003;71(1):483–93. Epub 2002/12/24. 12496199 10.1128/IAI.71.1.483-493.2003 PMC143283

[35] Radosevic K, Wieland CW, Rodriguez A, Weverling GJ, Mintardjo R, Gillissen G, et al Protective immune responses to a recombinant adenovirus type 35 tuberculosis vaccine in two mouse strains: CD4 and CD8 T-cell epitope mapping and role of gamma interferon. Infect Immun. 2007;75(8):4105–15. Epub 2007/05/29. 17526747 10.1128/IAI.00004-07 PMC1951991

[36] Auten MW, Huang W, Dai G, Ramsay AJ. CD40 ligand enhances immunogenicity of vector-based vaccines in immunocompetent and CD4+ T cell deficient individuals. Vaccine. 2012;30(17):2768–77. Epub 2012/02/22. 10.1016/j.vaccine.2012.02.020 22349523 PMC3313012

[37] Lee SE, Kim SY, Jeong BC, Kim YR, Bae SJ, Ahn OS, et al A bacterial flagellin, Vibrio vulnificus FlaB, has a strong mucosal adjuvant activity to induce protective immunity. Infect Immun. 2006;74(1):694–702. Epub 2005/12/22. 16369026 10.1128/IAI.74.1.694-702.2006 PMC1346682

[38] Bates JT, Uematsu S, Akira S, Mizel SB. Direct stimulation of tlr5+/+ CD11c+ cells is necessary for the adjuvant activity of flagellin. J Immunol. 2009;182(12):7539–47. Epub 2009/06/06. 10.4049/jimmunol.0804225 19494277 PMC3770462

[39] Schwarz K, Storni T, Manolova V, Didierlaurent A, Sirard JC, Rothlisberger P, et al Role of Toll-like receptors in costimulating cytotoxic T cell responses. Eur J Immunol. 2003;33(6):1465–70. Epub 2003/06/05. 12778463 10.1002/eji.200323919

[40] Seder RA, Darrah PA, Roederer M. T-cell quality in memory and protection: implications for vaccine design. Nat Rev Immunol. 2008;8(4):247–58. Epub 2008/03/08. 10.1038/nri2274 18323851

[41] Forbes EK, Sander C, Ronan EO, McShane H, Hill AV, Beverley PC, et al Multifunctional, high-level cytokine-producing Th1 cells in the lung, but not spleen, correlate with protection against Mycobacterium tuberculosis aerosol challenge in mice. J Immunol. 2008;181(7):4955–64. Epub 2008/09/20. 18802099 10.4049/jimmunol.181.7.4955 PMC2867031

[42] Kaufmann SH. Future vaccination strategies against tuberculosis: thinking outside the box. Immunity. 2010;33(4):567–77. Epub 2010/10/30. 10.1016/j.immuni.2010.09.015 21029966

[43] Abel B, Tameris M, Mansoor N, Gelderbloem S, Hughes J, Abrahams D, et al The novel tuberculosis vaccine, AERAS-402, induces robust and polyfunctional CD4+ and CD8+ T cells in adults. Am J Respir Crit Care Med. 2010;181(12):1407–17. Epub 2010/02/20. 10.1164/rccm.200910-1484OC 20167847 PMC2894413

[44] Murray PJ. Defining the requirements for immunological control of mycobacterial infections. Trends Microbiol. 1999;7(9):366–72. Epub 1999/09/02. 10470045 10.1016/s0966-842x(99)01567-x

[45] Kaufmann SH. Recent findings in immunology give tuberculosis vaccines a new boost. Trends Immunol. 2005;26(12):660–7. Epub 2005/10/26. 16246622 10.1016/j.it.2005.09.012

[46] Kowalczyk DW, Ertl HC. Immune responses to DNA vaccines. Cellular and molecular life sciences: CMLS. 1999;55(5):751–70. Epub 1999/06/24. 10379361 10.1007/s000180050330 PMC11147096

[47] Sallusto F, Lanzavecchia A, Araki K, Ahmed R. From vaccines to memory and back. Immunity. 2010;33(4):451–63. Epub 2010/10/30. 10.1016/j.immuni.2010.10.008 21029957 PMC3760154

[48] Bates JT, Honko AN, Graff AH, Kock ND, Mizel SB. Mucosal adjuvant activity of flagellin in aged mice. Mech Ageing Dev. 2008;129(5):271–81. Epub 2008/03/28. 10.1016/j.mad.2008.01.009 18367233 PMC2366812

[49] Caron G, Duluc D, Fremaux I, Jeannin P, David C, Gascan H, et al Direct stimulation of human T cells via TLR5 and TLR7/8: flagellin and R-848 up-regulate proliferation and IFN-gamma production by memory CD4+ T cells. J Immunol. 2005;175(3):1551–7. Epub 2005/07/22. 16034093 10.4049/jimmunol.175.3.1551

[50] Treanor JJ, Taylor DN, Tussey L, Hay C, Nolan C, Fitzgerald T, et al Safety and immunogenicity of a recombinant hemagglutinin influenza-flagellin fusion vaccine (VAX125) in healthy young adults. Vaccine. 2010;28(52):8268–74. Epub 2010/10/26. 10.1016/j.vaccine.2010.10.009 20969925

[51] Taylor DN, Treanor JJ, Sheldon EA, Johnson C, Umlauf S, Song L, et al Development of VAX128, a recombinant hemagglutinin (HA) influenza-flagellin fusion vaccine with improved safety and immune response. Vaccine. 2012;30(39):5761–9. Epub 2012/07/17. 10.1016/j.vaccine.2012.06.086 22796139

[52] Liaudet L, Murthy KG, Mabley JG, Pacher P, Soriano FG, Salzman AL, et al Comparison of inflammation, organ damage, and oxidant stress induced by Salmonella enterica serovar Muenchen flagellin and serovar Enteritidis lipopolysaccharide. Infect Immun. 2002;70(1):192–8. Epub 2001/12/19. 11748182 10.1128/IAI.70.1.192-198.2002 PMC127621

[53] Feghali CA, Wright TM. Cytokines in acute and chronic inflammation. Front Biosci. 1997;2:d12–26. Epub 1997/01/01. 9159205 10.2741/a171

[54] Hirano T. Interleukin-6 and Its Relation to Inflammation and Disease. Clinical immunology and immunopathology. 1992;62(1):S60–S5. 1728989 10.1016/0090-1229(92)90042-m

[55] Schumacher C, Clark-Lewis I, Baggiolini M, Moser B. High- and low-affinity binding of GRO alpha and neutrophil-activating peptide 2 to interleukin 8 receptors on human neutrophils. Proc Natl Acad Sci U S A. 1992;89(21):10542–6. Epub 1992/11/01. 1438244 10.1073/pnas.89.21.10542 PMC50375

[56] Dinarello CA. Reduction of inflammation by decreasing production of interleukin-1 or by specific receptor antagonism. International journal of tissue reactions. 1992;14(2):65–75. Epub 1992/01/01. 1399323

[57] Morris AE, Liggitt HD, Hawn TR, Skerrett SJ. Role of Toll-like receptor 5 in the innate immune response to acute P. aeruginosa pneumonia. Am J Physiol Lung Cell Mol Physiol. 2009;297(6):L1112–9. Epub 2009/10/06. 10.1152/ajplung.00155.2009 19801452 PMC2793188

[58] Tseng J, Do J, Widdicombe JH, Machen TE. Innate immune responses of human tracheal epithelium to Pseudomonas aeruginosa flagellin, TNF-alpha, and IL-1beta. Am J Physiol Cell Physiol. 2006;290(3):C678–90. Epub 2005/10/28. 16251478 10.1152/ajpcell.00166.2005

[59] Yang J, Zhong M, Zhang Y, Zhang E, Sun Y, Cao Y, et al Antigen replacement of domains D2 and D3 in flagellin promotes mucosal IgA production and attenuates flagellin-induced inflammatory response after intranasal immunization. Human vaccines & immunotherapeutics. 2013;9(5):1084–92. Epub 2013/02/05. 23377752 10.4161/hv.23809 PMC3899144

[60] Boog CJ. Principles of vaccination and possible development strategies for rational design. Immunol Lett. 2009;122(2):104–7. Epub 2008/12/23. 10.1016/j.imlet.2008.11.009 19100778

[61] Xiao Y, Liu F, Yang J, Zhong M, Zhang E, Li Y, et al Over-activation of TLR5 signaling by high-dose flagellin induces liver injury in mice. Cellular & molecular immunology. 2015 12 (6): 729–42. Epub 2014/11/25. 25418468 10.1038/cmi.2014.110 PMC4716618

[62] Rollier CS, Reyes-Sandoval A, Cottingham MG, Ewer K, Hill AV. Viral vectors as vaccine platforms: deployment in sight. Curr Opin Immunol. 2011;23(3):377–82. Epub 2011/04/26. 10.1016/j.coi.2011.03.006 21514130

[63] Geiben-Lynn R, Greenland JR, Frimpong-Boateng K, Letvin NL. Kinetics of recombinant adenovirus type 5, vaccinia virus, modified vaccinia ankara virus, and DNA antigen expression in vivo and the induction of memory T-lymphocyte responses. Clin Vaccine Immunol. 2008;15(4):691–6. Epub 2008/02/15. 10.1128/CVI.00418-07 18272665 PMC2292663

